# NF-κB-driven improvement of EHD1 contributes to erlotinib resistance in EGFR-mutant lung cancers

**DOI:** 10.1038/s41419-018-0447-7

**Published:** 2018-03-16

**Authors:** Xiaoyuan Wang, Hang Yin, Hongxia Zhang, Jing Hu, Hailing Lu, Chunhong Li, Mengru Cao, Shi Yan, Li Cai

**Affiliations:** 1 The Department of Internal Medical Oncology, Harbin Medical University Cancer Hospital, Harbin, China; 2 The Department of Radiotherapy Oncology, Harbin Medical University Cancer Hospital, Harbin, China; 3 The Department of MRI, Harbin Medical University Cancer Hospital, Harbin, China

## Abstract

Acquired resistance to epidermal growth factor receptor-tyrosine-kinase inhibitors (EGFR-TKIs), such as gefitinib and erlotinib, is a critical obstacle in the treatment of EGFR mutant-positive non-small cell lung cancer (NSCLC). EHD1, a protein of the C-terminal Eps15 homology domain-containing (EHD) family, plays a role in regulating endocytic recycling, but the mechanistic details involved in EGFR-TKI resistance and cancer stemness remain largely unclear. Here, we found that a lower EHD1 expression improved both EGFR-TKIs sensitivity, which is consistent with a lower CD133 expression, and progression-free survival in NSCLC patients. The overexpression of EHD1 markedly increased erlotinib resistance and lung cancer cell stemness in vitro and in vivo. Moreover, we demonstrated that miR-590 targeted the 3′-UTR of EHD1 and was regulated by NK-κB, resulting in downregulated EHD1 expression, increased erlotinib sensitivity and repressed NSCLC cancer stem-like properties in vitro and in vivo. We found that EHD1 was an important factor in EGFR-TKI resistance and the cancer stem-like cell phenotype of lung cancer, and these results suggest that targeting the NF-κB/miR-590/EHD1 pathway has potential therapeutic promise in EGFR-mutant NSCLC patients with acquired EGFR-TKI resistance.

## Introduction

Lung cancer is the leading cause of cancer-related deaths worldwide^1^. Small-molecule tyrosine-kinase inhibitors (TKIs) are successfully used for patients harbouring epithelial growth factor receptor (EGFR)-activating mutations in non-small cell lung cancer (NSCLC), such as gefitinib and erlotinib^2,3^. Despite an initial response to EGFR-TKIs, NSCLC patients with EGFR-TKI treatment eventually become resistance after 10–16 months, leading to treatment failure. The primary mechanisms of acquired resistance are ascribed to the secondary EGFR mutation T790M in exon 20, MET oncogene amplification, small-cell carcinoma transformation, epithelial-mesenchymal transition, HER2 amplification, and minor subpopulations of cancer stem cells (CSCs)^4–7^. However, further investigations of CSC-like properties and their role in the treatment of drug-resistant lung cancer are needed.

C-terminal Eps15 homology domain-containing (EHD) proteins are a family of highly conserved proteins involved in regulating endocytic recycling^8^. This family consists of four highly homologous members in mammalian cells (EHD1–4)^9^. These proteases are abnormally expressed in various solid tumours with poor prognosis, including head and neck, lung, and breast cancer^10–13^. EHD1 has also been shown to play a role in recycling receptors from early endosomes into the endocytic recycling compartment (ERC) and from the ERC to the plasma membrane. Importantly, EHD1 regulates β1 integrin endosomal transport, significantly affecting proliferation, focal adhesions, apoptosis, migration, and metastasis^14–16^. Moreover, EHD1 is also involved in vesicle trafficking, which is associated with cancer migration, metastasis, and poor patient survival in NSCLC^17,18^. Although the aberrant expression of EHD1 has been reported in various diseases, whether this protein regulates CSC properties remains unclear.

Here, we found that EHD1 is an important factor in EGFR-TKI resistance and the cancer stem-like cell phenotype of lung cancer, which suggests that targeting the NF-κB/miR-590/EHD1 pathway offers potential therapeutic promise for NSCLC patients with EGFR-mutations and acquired EGFR-TKI resistance.

## Methods

### Cell culture

The NSCLC cell lines PC9/GR, PC9, H1650, HCC827, H1975, H1299, H1666, NCI-H358, A549, WI38, H460, and HBE were obtained from Heilongjiang Cancer Institute (Harbin, China). Cells were cultured in RPMI (Sigma-Aldrich, Madrid, Spain) with 10% foetal bovine serum and then incubated at 37 °C in a humidified atmosphere with 5% CO_2_.

### Immunohistochemistry

Lung cancer specimens were obtained from the Harbin Medical University Cancer Hospital, and each patient signed an informed consent forms for medical record review and tissue sample donation. Between December 2008 and June 2010, 23 patients with stage IV EGFR-mutant NSCLC were enroled into this retrospective study. Based on their response to TKI, patients were divided into two groups: EGFR-TKI nonresponders (*n* = 11) and EGFR-TKIs responders (*n* = 12). EGFR-TKI nonresponders (*n* = 11) were patients who had progressive disease or stable disease without durable (6 months) poor progression-free survival (PFS). EGFR-TKIs responders (*n* = 12) were patients who had a complete response, partial response, or stable disease status with prolonged PFS (12 months)^19^. Immunohistochemistry assays were performed on the paraffin-embedded NSCLC tissue^20^. The slides were incubated with EHD1 (Abcam, #ab109311, diluted at 1:100) or CD133 (JKSJ-orb372326, diluted at 1:100) primary antibody. The relative staining intensity was defined as 0 when 5% of the tumour cells were positively stained; weak (1) for 5–25% staining; moderate (2) for 25–50%; and strong (3) for 50%. A score ≤1 was classified as low expression, and a score ≥2 was classified as high expression.

### Cell proliferation

Cells were plated in a 96-well plate in triplicate at 1 × 10^3^ cells per well and cultured with or without TKI at the indicated concentrations and for the specified durations. At the end of each time point, CCK-8 was added for 1 h at 37 °C, and the absorbance at 450 nm was detected.

### Western blot analysis

Western blot analysis was performed according to a previously described standard method^21^. Proteins were probed with EHD1 (Abcam, #ab109311, diluted at 1:1000) or P50 (Abcam, #ab32360, diluted at 1:1000) antibodies. The bound antibodies were detected using an ECL Western Blotting Detection system. The blotted membranes were reblotted with β-actin (Sigma, A1978) as the loading control.

### Real-time PCR

Total cellular RNA from the cultured cells was isolated using TRIzol reagent (Invitrogen). RNA samples were then reverse-transcribed into cDNA using a cDNA Synthesis Kit (Fermentas, Foster City, CA). RNA expression levels were determined by a SYBR Premix Ex-Taq II Kit and an ABI 7500 machine and were then normalised to β-actin using the 2^−ΔΔI^ method. All procedures were performed following the manufacturer’s instructions.

### Plasmid transfection

ShRNA-EHD1 plasmids or control plasmids were purchased from GenePharma. The sequences for EHD1 were as follows: 5′-GCCUGCAUGAAGUCUGUAATTUUACAGACUUCAUGCAGGCTT-3′. The PLKO vector was used. Infected cells were selected by puromycin (1.25 µg/ml). EHD1-OE, miR-590 mimics and control, inhibitor and negative control, PENTER vector (NF-κB-NC) and NF-κB overexpression vectors (NF-κB-OE) were mixed with Lipofectamine 2000 (Invitrogen) and then added to cell culture medium according to the manufacturer’s instructions.

### Sphere formation assay

Cells (1000 cells/ml) were suspended in serum-free DMEM/F12 (Invitrogen-Gibco, Paisley, UK) supplemented with B27 (1:50, Gibco), 20 ng/ml epidermal growth factor (Prospec), 5 mg/ml insulin (Sigma), and 0.4% BSA (Sigma). After 10–12 days of culture, the tumourspheres were analysed and quantified using a microscope (Olympus).

### Surface marker analysis by flow cytometry

Cells were washed with PBS, stained with anti-CD133 (Miltenyi Biotec, CD133/1 (AC133)-PE, 5151001186) antibody in PBS containing 1% FBS and incubated on ice for 30 min in the dark. The cells were washed again with cold PBS, and >10,000 cells were analysed by flow cytometry using a BD Canto II flow cytometer (BD Biosciences). The data were analysed using FACSDiva software (BD Biosciences).

### Dual luciferase reporter assay

Cells (1 × 10^5^ cells/well) were seeded into 24-well plates and then transfected with EHD1-3′-UTR-wt or EHD1-3′-UTR-mt and miR-590 using Lipofectamine 2000 (Invitrogen) according to the manufacturer’s protocol. At 24 h after transfection, a Dual Luciferase Reporter Assay System (Promega) was used to measure luciferase activity, and the activity was normalized to that of Renilla luciferase.

### Xenograft models

Animal experiments were approved by the Scientific Investigation Board of Tumours Hospital of Harbin Medical University. Cells (1 × 10^6^) were implanted into male BALB/c nude mice (Beijing Vital River Laboratory Animal Technology Co., Ltd.). Anti-agomir-miR-99a or anti-agomir-NC (RiboBio Co., Ltd., Guangzhou, China) was directly injected into the implanted tumour at a dose of 1 nmol/mouse every 4 days and was repeated seven times. Tumour volume (*V*) was calculated with the following equation: *V* = (*W*^2^ × *L*) × 0.5. When tumour volumes reached ∼100 mm^3^, the mice were randomly allocated into groups to receive erlotinib or vehicle at 100 mg/kg by oral gavage every day.

### Statistical analysis

SPSS 18.0 and GraphPad Prism software were used for statistical analyses. The statistical significance between two groups was calculated by a two-tailed Student’s *t*-test. Survival curves were plotted using the Kaplan–Meier method and were compared between the groups using the log-rank test. *P*-values less than 0.05 were considered statistically significant (*).

## Results

### EHD1 expression is associated with EGFR-TKI resistance and CSC-like properties

**Fig. 1:**
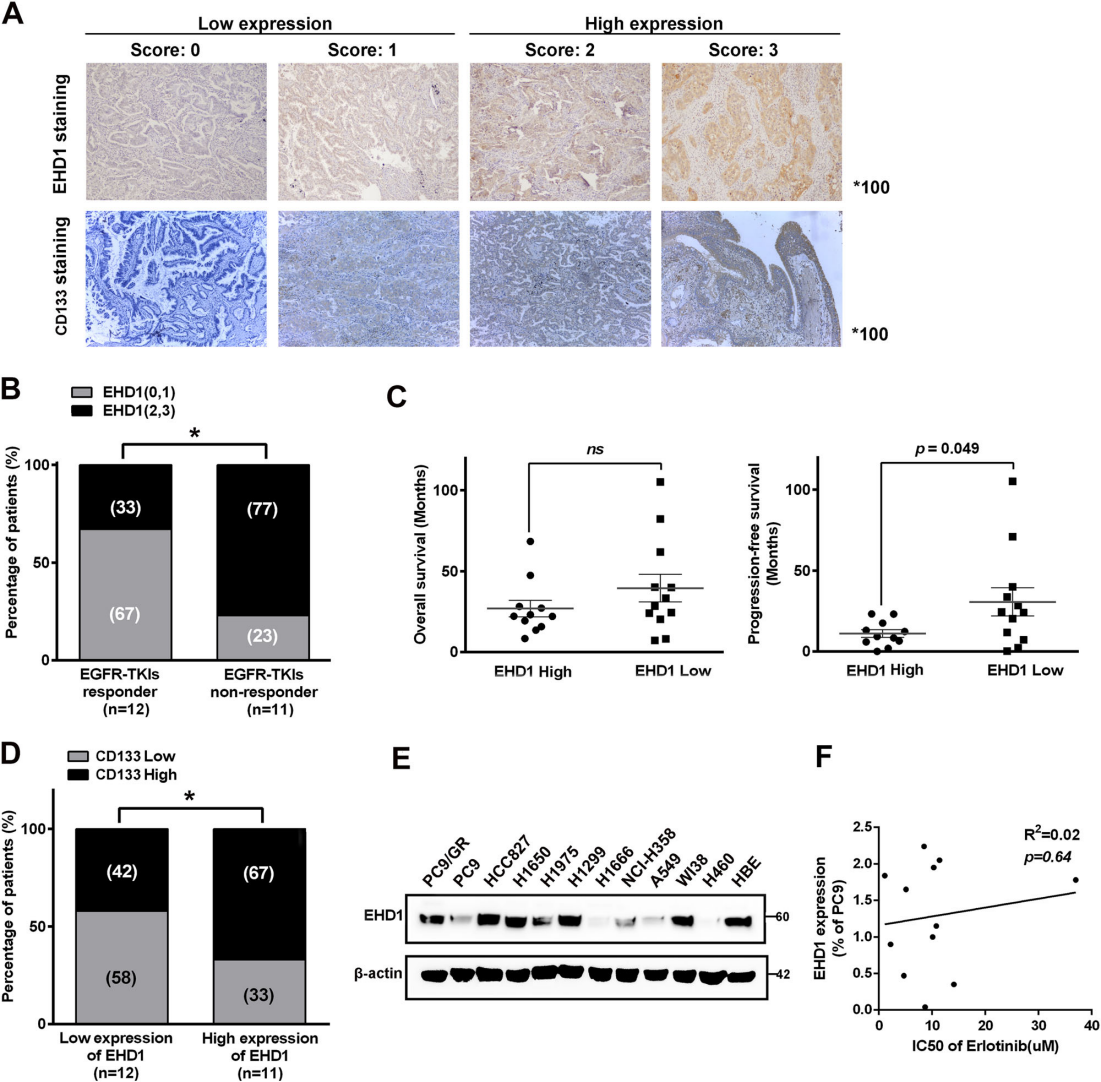
EHD1 expression is associated with the response to erlotinib treatment and stemness in lung cancer. **a** IHC staining yielded scores ranging from 0 to 3, which are representative of the amount of EHD1 and CD133 in lung cancer specimens. A score of 0–1 represented low expression, and 2–3 represented high expression. **b** The percentages of patients with high expression (black bar) and low expression of EHD1 (grey bar) were assigned according to different responses to EGFR-TKI (responder, *n* = 12; nonresponder, *n* = 11). Numbers near bars represent the percentage of patients for each condition. *P < 0.05. **c** Mean OS of patients with high levels of EHD1 (*n* = 11, OS of 26 mo) and low levels of EHD1 (*n* = 12, OS of 39 mo) taking EGFR-TKI treatment. Mean PFS of patients with high levels of EHD1 (*n* = 11, PFS of 11 mo) and low levels of EHD1 (*n* = 12, PFS of 30 mo) taking EGFR-TKI treatment. *P* = 0.049. **d** Patients with high CD133 expression were accompanied by increased expression of EHD1 protein. The expression levels of EHD1 were classified into two groups according to the IHC scoring: low-expression group (score 0 and 1) and high-expression group (score 2 and 3). **e** EHD1 expression in human lung cancer cell lines. **f** The correlation between EHD1 expression and IC50 of erlotinib in human lung cancer cell lines

To elucidate the relationship between EHD1 expression and EGFR-TKI resistance, we retrospectively evaluated specimens from a total of 24 lung cancer patients who had received EGFR-TKIs (erlotinib or gefitinib). By using IHC staining, we detected the expression of EHD1 and found that the cytoplasmic staining intensity of EHD1 was stronger than the nuclear staining (Fig. 1a), and the high-EHD1 expression was identified in 47.8% of the lung cancer samples (*n* = 23). As shown in Fig. 1b, EHD1 overexpression was more frequently observed in patients with a poor response to EGFR-TKIs therapy than in patients who responded to EGFR-TKI treatment. Moreover, EHD1 expression was found to be closely correlated with PFS in patients (11.05 m vs. 30.71 m, *P* < 0.05, Fig. 1c). To investigate the correlation between EHD1 expression and stem cell-like properties in NSCLC patients, the expression of CD133 was detected. EHD1 expression was positively correlated with CD133 expression (Fig. 1a, d). A significant decrease in EHD1 expression was observed in parental PC9/WT cells compared to erlotinib-resistant PC9 (PC9/GR) cells containing the same exon 19 deletion of EGFR, as detected by western blotting (Fig. 1e). These results suggest that EHD1 was positively correlated with TKI resistance and CSC-like properties in lung cancer.

### Effects of EHD1 involved in erlotinib resistance and CSC properties

**Fig. 2:**
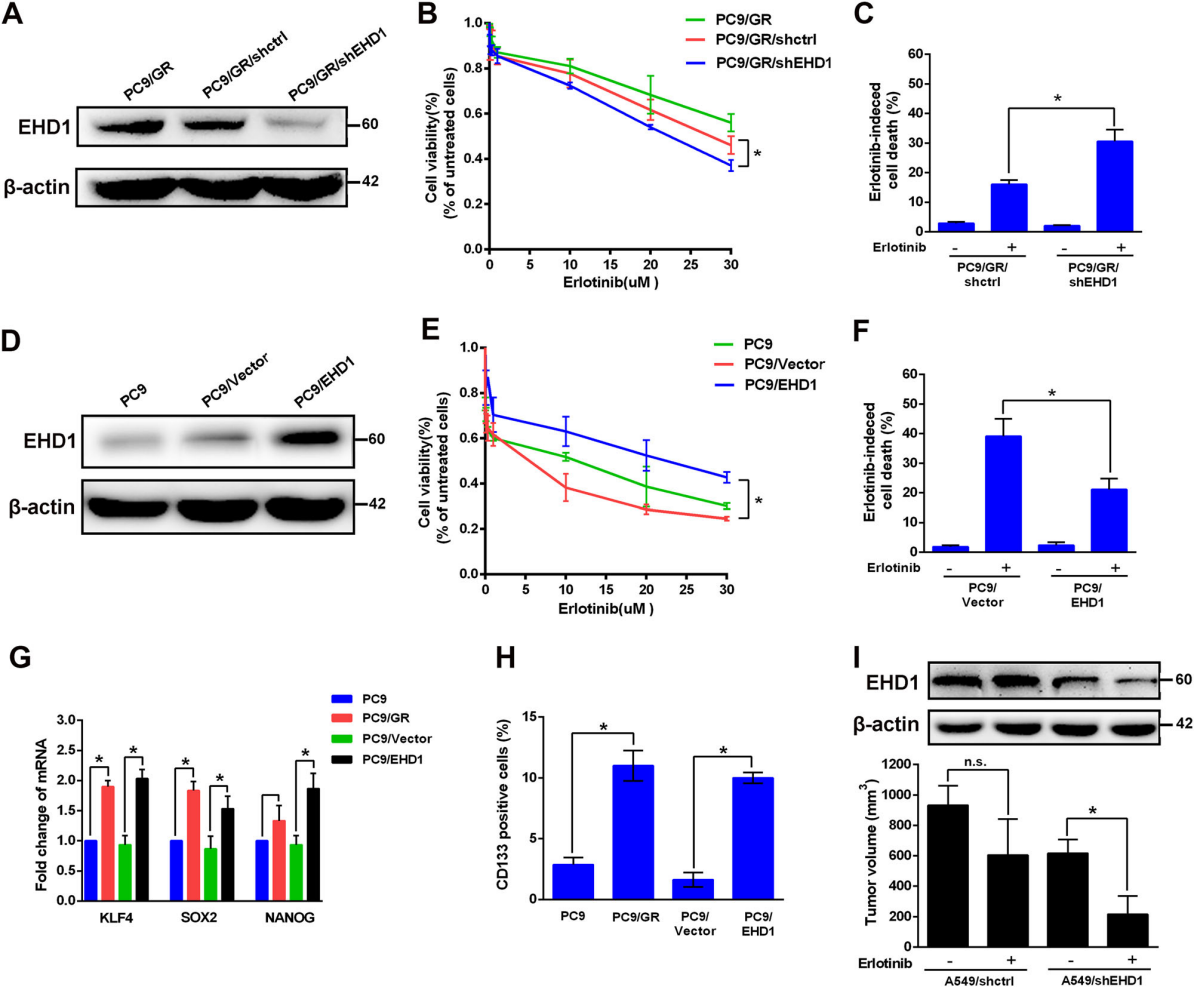
EHD1 is required for erlotinib-induced cell death and CSC markers of lung cancer cells. **a** EHD1 expression was analysed by western blot in PC9/GR, PC9/GR/shctrl, and PC9/GR/shEHD1 cells. **b** PC9/GR/shEHD1 and corresponding vector control cells were treated with erlotinib, and cell viability was measured by CCK assay and **c** with erlotinib cell apoptosis was detected by flow cytometric analysis, respectively. **d** Western blot analysis of EHD1expression in PC9, PC9/vector, and PC9/EHD1 cells. **e** The indicated concentrations of erlotinib were added to these cells; cell viability was measured by CCK assay, and **f** cell apoptosis was detected by flow cytometric analysis.** g** The effects of EHD1 expression mediated mRNA expression of KLF4, SOX2, and Nanog. Total RNA was harvested for the analysis of mRNA by real-time RT-PCR. **h** The effects of EHD1 expression mediated CD133+ cells. Cells were harvested for flow cytometric analysis to detect CD133+ cells. The results are shown as the means ± SD of three independent experiments, each performed in triplicate. *P < 0.05. **i** Effects of EHD1 depletion on the tumour growth of A549 cells were evaluated in the NOD/SCID mice with xenografts after treatment with erlotinib for 2 weeks. *P < 0.05

To examine the role of EHD1 in erlotinib resistance, we established an EHD1-knockdown PC9/GR cell line (Fig. 2a) and showed that PC9/GR/sh-EHD1 cells were significantly more sensitive to erlotinib (Fig. 2b) and had increased more apoptotic cells (16.07 ± 0.84% vs. 30.06 ± 2.31%, *P* < 0.05, Fig. 2c). In addition, PC9 cells overexpressing EHD1 (PC9/EHD1) (Fig. 2d) were more resistant to erlotinib (Fig. 2e) and had decreased erlotinib-induced apoptosis compared to the PC9/vector cells and (39.10 ± 3.44% vs. 21.17 ± 2.08%, *P* < 0.05, Fig.2f) which was consistent in A549 cells (SI Appendix, Fig. S1). PC9/GR cells exhibited significantly higher levels of CSC markers, such as Klf4, Sox2, Nanog, and CD133+ cells, than the PC9 cells, and these genes were upregulated in the PC9/EHD1 group compared to the PC9/vector group (Fig. 2g, h). In addition, after erlotinib treatment, the A549/shEHD1-bearing groups had a lower average tumour volume than the A549/shctrl-bearing group (Fig. 2i). These data indicated that EHD1 plays a critical role in erlotinib responsiveness and NSCLC cell stemness.

### MiR-590 targeting of EHD1 is correlated with erlotinib sensitivity

**Fig. 3:**
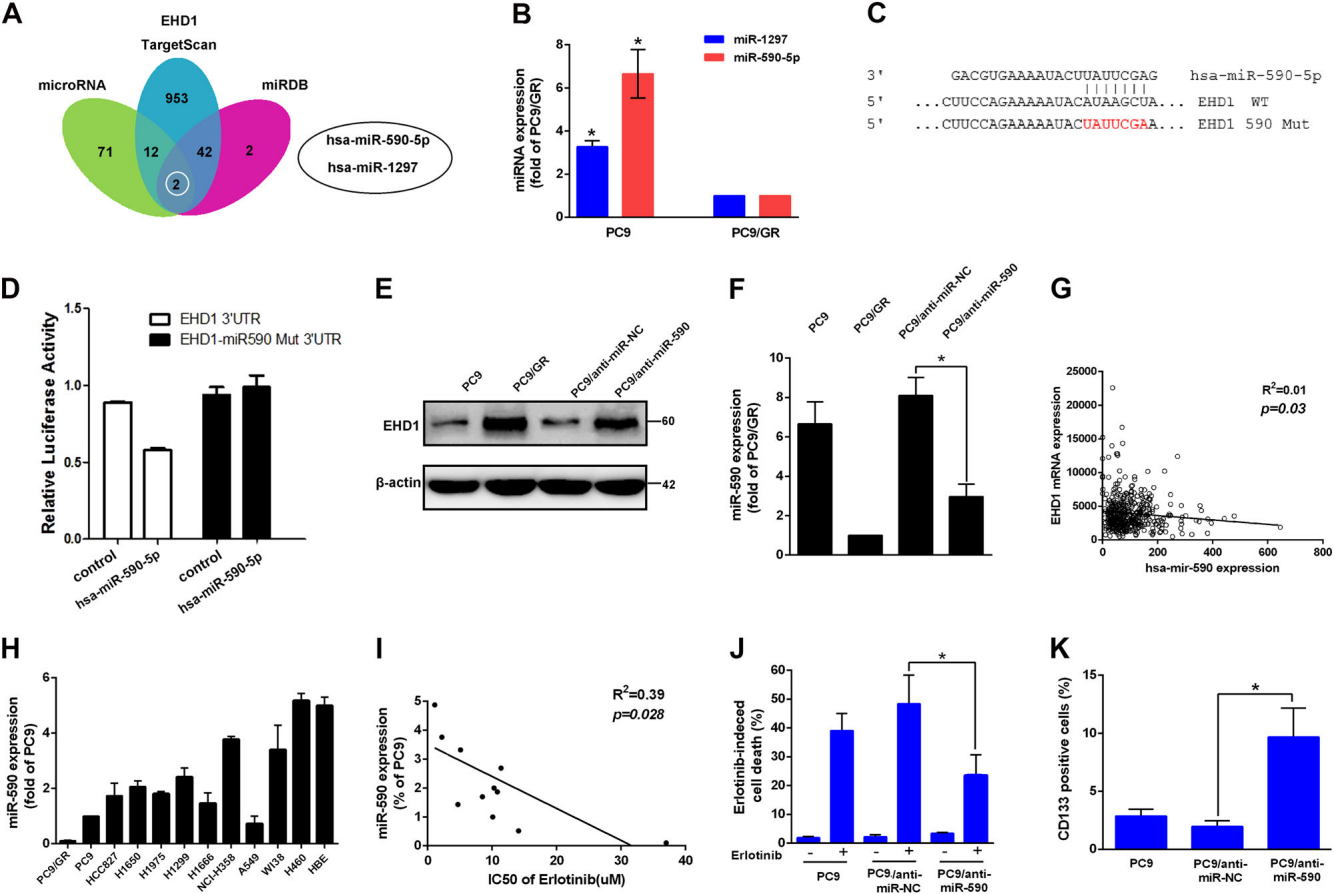
MiR-590 targeting of EHD1 is correlated with erlotinib sensitivity. **a** Schematic of the bioinformatic analyses of candidate microRNAs that targeted EHD1. The putative microRNAs targeting EHD1 in microRNA, TargetScan and miRDB were searched and two candidate microRNAs were filtered out for real-time RT-PCR analysis. **b** Real-time RT-PCR analysis of the differential expression of the two microRNAs in PC9 compared with PC9/GR cells. **c**, **d** A schematic diagram representing the predicted miR-590 binding sequences or mutated versions (upper). Luciferase activity (lower) of wild-type EHD1–3′ UTR (EHD1–3′ UTR) and mutant-type EHD1–3′ UTR (Mut-3′ UTR) reporter genes were measured using the Luciferase Reporter Assay in PC9/WT cells transfected with miR-590. **e**, **f** The effects of miR-590 knockdown on EHD1 protein expression in PC9/GR cells. EHD1 and miR-590 expression were measured by western blot analysis and real-time RT-PCR, respectively. **g** The inverse correlation between relative miR-590 expression and EHD1 mRNA expression in NSCLC tissues from patients according to the TCGA database, *n* = 445; *r*^2^ = 0.01; *P* = 0.03. **h** MiR-590 expression in human lung cancer cell lines. **i** The correlation between miR-590 expression and the IC50 of erlotinib with doses for the lung cancer cell lines (*r*^2^ = 0.39; *P* = 0.028; Pearson’s correlation coefficient). **j** The functions of miR-590 in regulating erlotinib sensitivity were assayed by comparing PC9/anti-miR-NC and PC9/anti-miR-590 that were treated with or without erlotinib. Cell viability was determined by CCK assay. **k** CD133+ cells by flow cytometric analysis in PC9/anti-miR-NC and PC9/anti-miR-590 cells. *P < 0.05

To identify whether EHD1 was regulated by microRNAs (miRNAs), three algorithmic methods were used. After analysing the miRNA targets predicted by miRNA, TargetScan and miRDB, two candidate miRNAs localised to the three main centres predicted to target the EHD1 3′-UTR (Fig. 3a). Then, we determined the levels of miR-590 and miR-1297 in PC9 and PC9/GR cells and found that miR-590 was more significantly increased in PC9 cells than in PC9/GR cells (6.59 ± 1.02 fold, *P* < 0.05, Fig. 3b). To further evaluate whether miR-590 directly regulates EHD1, the wild-type EHD1 3′-UTR or mutant EHD1 3′-UTR was cotransfected with either has-miR-590-5p mimics or the negative control into 293T cells. We found that the wild-type 3′-UTR reporter significantly inhibited luciferase activity, and this effect was not observed with the mutant constructs (Fig. 3c, d). Western blot analysis further confirmed that the downregulation of miR-590 markedly increased the protein expression of EHD1 (Fig. 3e). A linear regression analysis of NSCLC tissues from patients in the TCGA database also indicated an inverse correlation between the relative miR-590 expression and EHD1 mRNA expression (*r*^2^ = 0.01, *P* = 0.03, Fig. 3g). Moreover, we found there is an inverse correlation between miR-590 expression and erlotinib resistance in lung cancer cells (*r*^2^ = 0.39, *P* = 0.03, Fig. 3h, i). PC9 cells with anti-miR-590 had markedly increased erlotinib resistance (48.40 ± 5.76% vs. 23.53 ± 4.13%, *P* < 0.05, Fig. 3j) and CD133+ cells compared with PC9/anti-NC cells (Fig. 3k). These results illustrated that miR-590 directly regulated EHD1 expression and that decreased miR-590 expression resulted in poor EGFR-TKI sensitivity and a CSC phenotype.

### MiR-590 represses EHD1-mediated erlotinib resistance

**Fig. 4:**
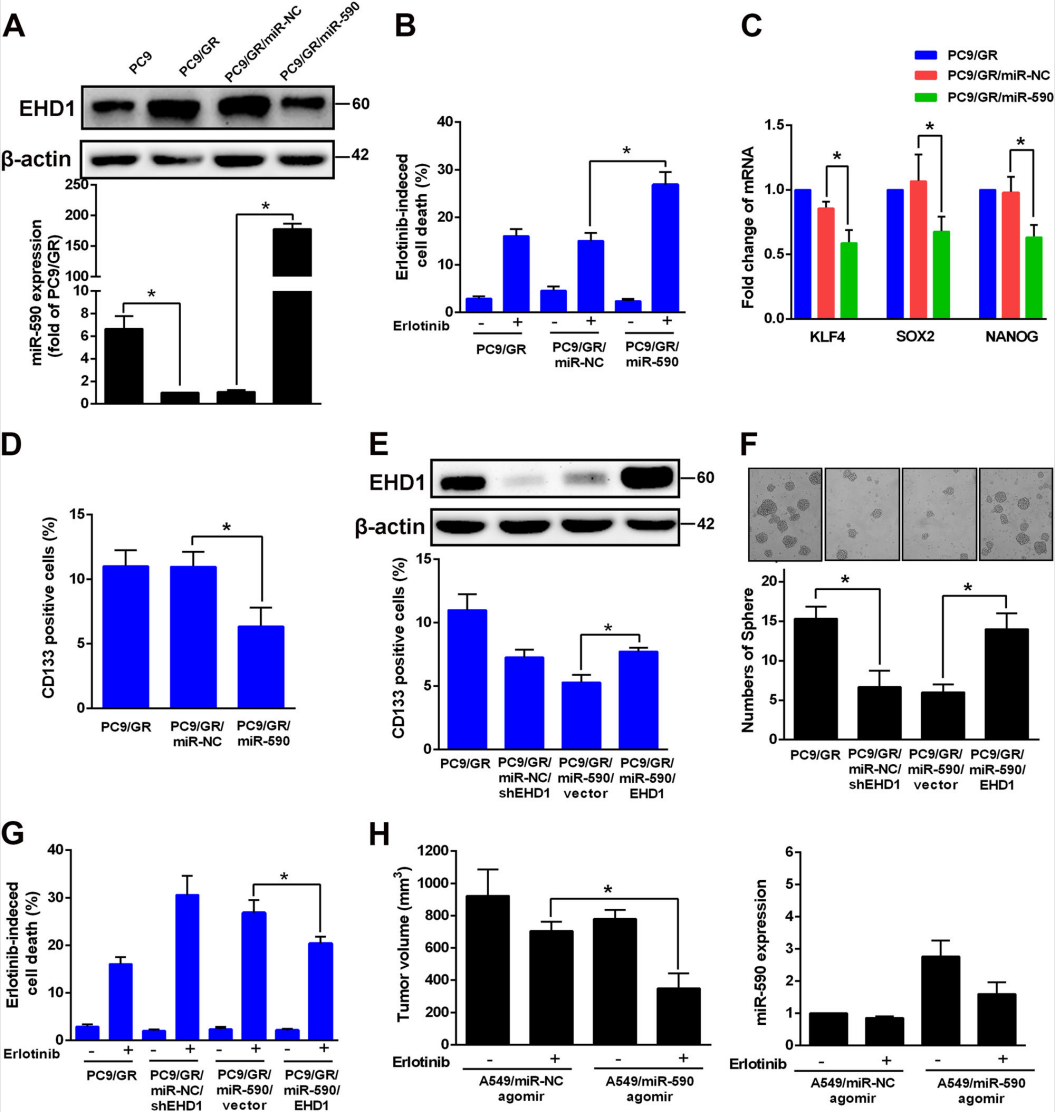
MiR-590 represses EHD1-mediated erlotinib resistant and CSC characteristics. **a** miR-590 was measured by real-time RT-PCR in PC9/GR, and PC9/GR cells with expressing miR-590 (PC9/GR/miR-590) and PC9/GR/miR-NC, respectively. **b** Indicated cells were untreated or treated with erlotinib and analysed for cell viability by CCK assay. **c** The effects of miR-590 expression mediated mRNA expression of KLF4, SOX2 and Nanog. **d** The effects of miR-590 expression mediated CD133+ cells. **e** The effects of transfection EHD1 in miR-590-overexpressing PC9/GR cells on CSC phenotypes. **f** The ability to form spheres and **g** erlotinib sensitivity. **h** Effects of miR-590 overexpression with or without transfection EHD1 on the tumour growth of PC9/GR cells were evaluated in the NOD/SCID mice with xenografts after treatment with erlotinib for 2 weeks. *P < 0.05

Next, the role of miR-590-repressed EHD1-mediated erlotinib resistance and stemness was further examined. PC9/GR cells were transfected with miR-NC or miR-590 mimics, and the infection efficiency was confirmed by RT-PCR (Fig. 4a). Compared with control cells, PC9/GR cells with high miR-590 levels showed increased erlotinib-induced cell death (15.07 ± 0.95% vs. 26.87 ± 1.54%, *P* < 0.05, Fig. 4b) and decreased CSC properties and CD133+ cells (Fig. 4c, d). Next, we transfected EHD1-expressing constructs and vectors into miR-590-overexpressing PC9/GR cells. As shown in Fig. 4e, f, g, EHD1 transfection in miR-590-overexpressing PC9/GR cells successfully abolished the miR-590-mediated CSC phenotype, the ability of cells to form spheres and erlotinib sensitivity. The in vivo results were consistent with the observations in vitro: tumours treated with the miR-590 agomir were more sensitive to erlotinib treatment than the miR-NC agomir-treated tumours (704.70 ± 32.99 mm^3^ vs. 348.70 ± 53.72 mm^3^, *P* < 0.05, Fig. 4h) because EHD1 expression was low. These experiments revealed that overexpressing miR-590 improves cell responsiveness to EGFR-TKIs and decreases CSC stemness in vivo and in vitro, and this is accomplished via the regulation of EHD1 by miR-590.

### NF-κB participates in the regulation of miR-590 and erlotinib sensitivity

**Fig. 5:**
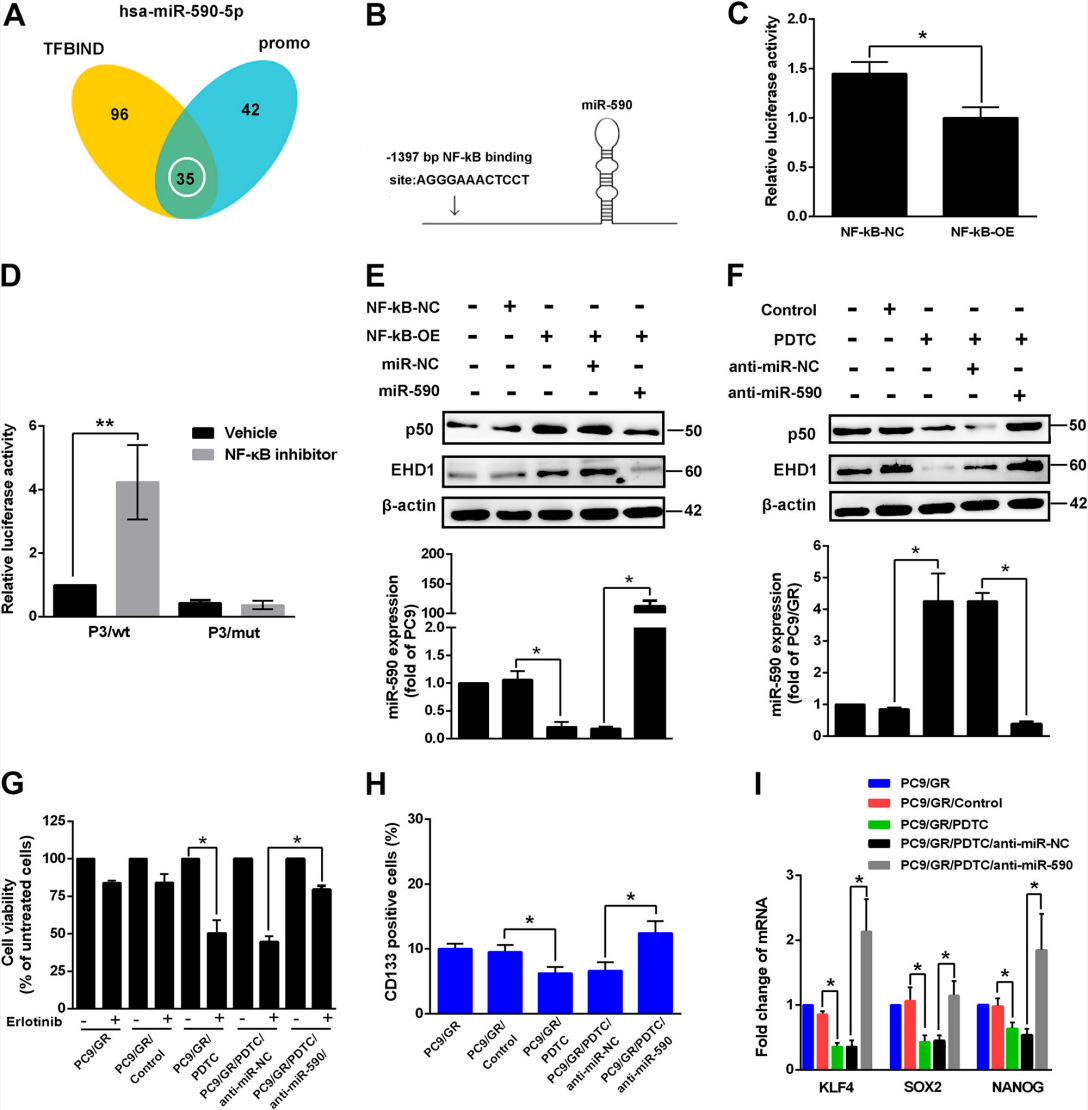
Inhibition of NF-κB activity results in increased miR-590 expression and suppressed erlotinib resistance and CSC properties. **a** Schematic of the bioinformatic analyses of predicted transcription factors that targeted miR-590. NF-κB was one of 35 predicted genes were filtered out by the search programs of TFBIND and PROMO. **b** Schematic diagram showing that the NF-κB binding sequences in the miR-590, and **c** luciferase activity was measured using the luciferase reporter assay. **d** Reporter assays of miR-590 promoter containing wild-type or mutant NF-κB binding site cloned into the pGL3-Basic vector in PC9 cells treated with NF-κB inhibitor. **e**, **f** The EHD1 and p50 protein expression of cells with stable expression of the indicated transfectants were measured by western blot (upper), and miR-590 expression by real-time RT-PCR (lower), respectively. *P < 0.05. **g**, **h** Indicated cells were untreated or treated with erlotinib and NF-κB inhibitor, and analysed for cell viability by CCK assay and CD133+ cells by flow cytometric analysis. *P < 0.05. **i** Expression of NF-κB affected mRNA levels of KLF4, SOX2 and Nanog by real-time RT-PCR

The expression of miRNA, including primary miRNA transcription, maturation and degradation, is regulated by transcription factors. To identify the transcription factors involved in the regulation of miR-590 expression, two bioinformatics methods were used. By analysing predicted transcription factors by the search programmes TFBIND and PROMO, NF-κB was the candidate gene that localized at the two main network centres composed of 35 predicted genes (Fig. 5a). Putative binding sites for NF-κB were identified (Fig. 5b). To investigate the involvement of this transcription factor in miR-590 gene regulation, we examined miR-590 luciferase activity in PC9 cells by luciferase reporter assay using NF-κB-OE and control vectors (Fig. 5c). The results showed that the overexpression of NF-κB maintains a lower miR-590 promoter activity than that of the control cells, resulting in increased EHD1 expression (Fig. 5c–e). Moreover, a reporter assay was used by the transfection of the miR-590 promoter construct containing a wild-type or mutant NF-κB binding site in PC9 cells. We observed a significantly increased promoter activity of wildtype miR-590 reporter but not the mutant miR-590 reporter after treatment with the NF-κB inhibitor pyrrolidine dithiocarbamate (PDTC) (Fig. 5d). Next, we found that PC9 cells treated with PDTC had increased miR-590 expression and decrease EHD1 expression, but these effects were reversed in cells with si-miR-590 (Fig. 5f). Next, we wondered whether inhibiting NF-κB activity regulates cell sensitivity to EGFR-TKIs. Fig. 5g–i shows that treatment with PDTC resulted in decreased EHD1 expression, increased erlotinib sensitivity and reduced CSC properties in PC9/GR cells. The results from inhibiting NF-κB were reversed by the treatment of PC9/GR/PDTC cells with si-miR-590. These findings suggest that NF-κB binds to miR-590, reduces miR-590 expression and then increases EHD1 expression, leading to increased CSC stemness and erlotinib resistance.

### EHD1 is correlated with EGFR-TKI sensitivity and patient survival in NSCLC

**Fig. 6:**
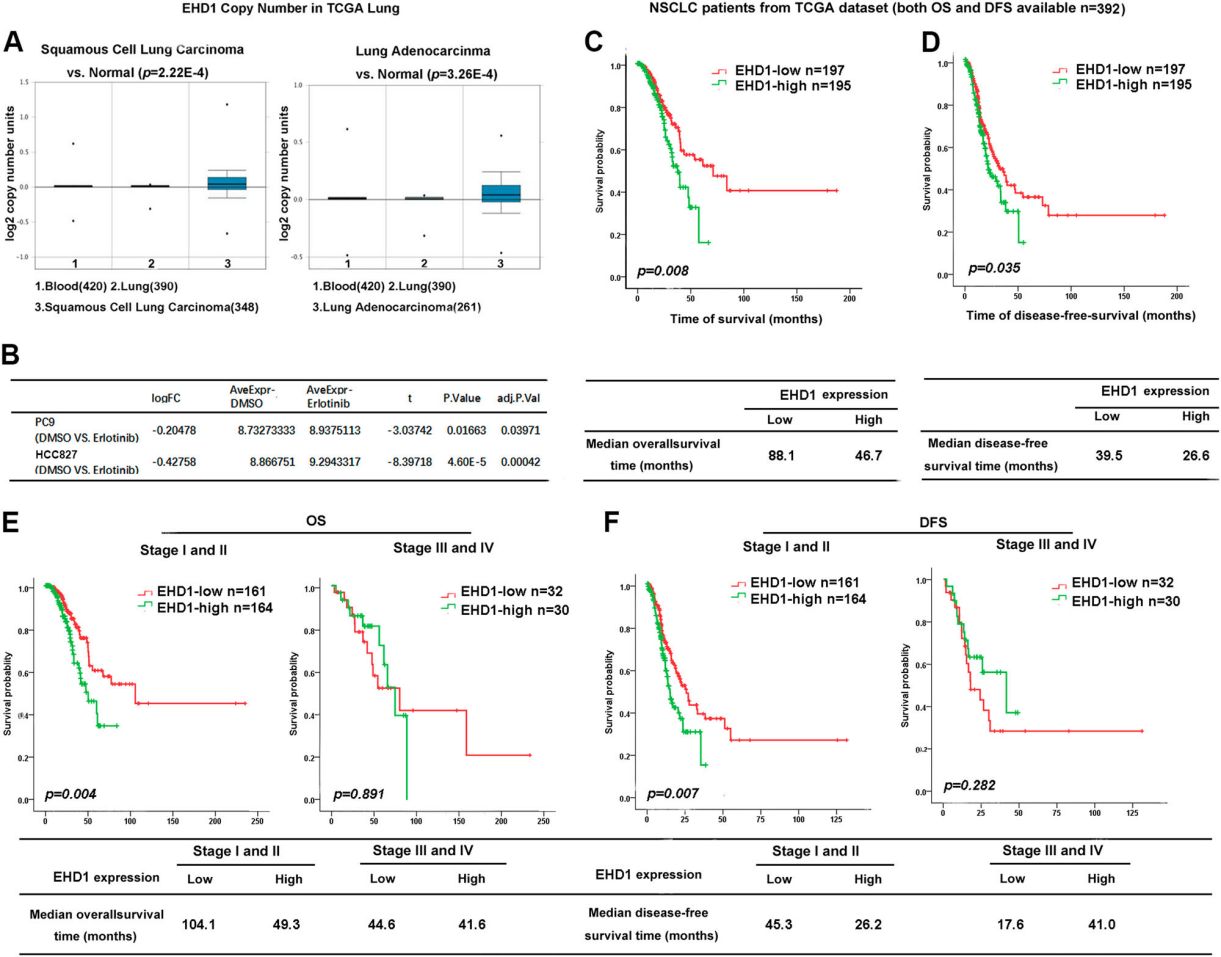
EHD1 correlates with EGFR-TKI sensitivity and patient survival in NSCLC. **a** The EHD1 expression in squamous cell lung carcinoma samples, lung adenocarcinoma samples and para-carcinoma normal lung tissues obtained from The Cancer Genome Atlas (TCGA) database. **b** The expression of EHD1 in PC9 and HCC827 cells treated with EGFR-TKI or with DMSO from TCGA. **c**, **d** Kaplan–Meier analysis of the 5-year OS (left panel) and DFS (right panel) of patients with NSCLC for whom both OS and DFS information was available in the TCGA lung cancer data set. The patients were stratified by high (greater than the median, *n* = 195) vs. low (greater than or equal to the median, *n* = 197) expression of EHD1. **e**, **f** Further analyse OS and DFS for 325 NSCLC patients in stages I–II and 62 patients in stages III–IV in the TCGA lung cancer cohort, respectively

To determine the role of EHD1 in NSCLC development, we analysed EHD1 expression in squamous cell lung carcinoma samples, lung adenocarcinoma samples and para-carcinoma normal lung tissues obtained from The Cancer Genome Atlas (TCGA) database and found that EHD1 expression were dramatically upregulated in both adenocarcinomas and squamous cell carcinomas compared with the non-malignant samples (*P* < 0.01, Fig. 6a). The expression of EHD1 was increased in PC9 and HCC827 cells treated with EGFR-TKIs compared with DMSO-treated cells from TCGA (*P* < 0.01, Fig. 6b). To identify whether EHD1 is relevant to NSCLC patient survival, EHD1 expression was analysed in the NSCLC patients with available DFS and OS data in the lung data set from TCGA (*n* = 392). The median OS for the high and low EHD1-expressing groups were 46.7 and 88.1 months (*P* = 0.008), respectively, and the median DFS was 26.5 and 39.5 months (*P* = 0.035, Fig. 6c, d). Likewise, in TCGA lung cancer samples, patients in early stages (stages I and II) with low EHD1 expression had a longer OS and DFS than those with high EHD1 expression (*P* = 0.004 and 0.007, respectively), indicating that EHD1 level may indicate NSCLC patient prognosis during early clinical stages, although there was no significant difference in the OS and DFS of advanced patients (Fig. 5e, f). Collectively, our results demonstrated that EHD1 plays an important role in EGFR-TKI sensitivity and prognosis in NSCLC patients.

### NF-κB/MiR-590/EHD1 regulates erlotinib response in vivo

**Fig. 7:**
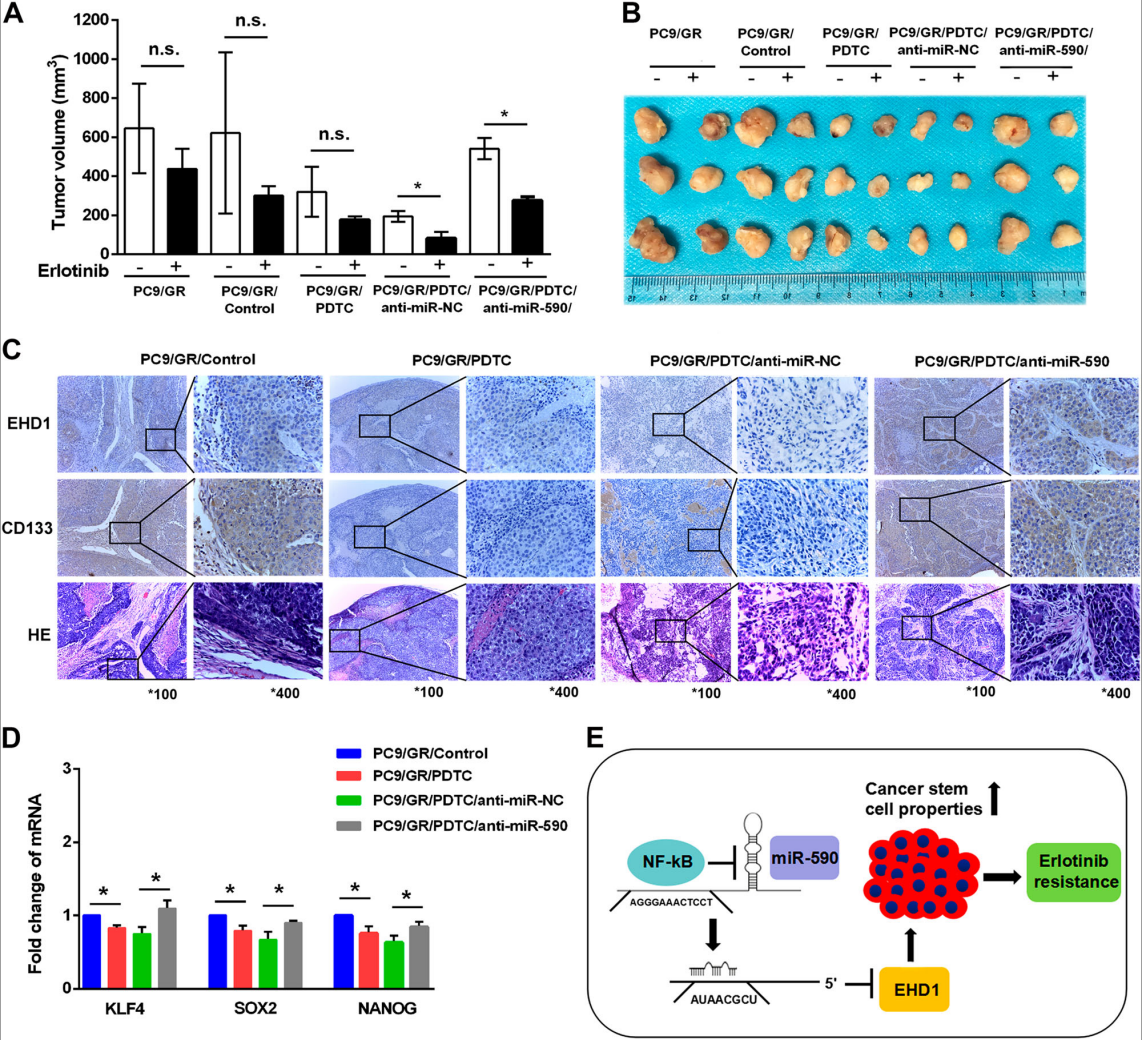
NF-κB/miR-590–mediated EHD1 suppression is required for erlotinib resistance and stemness of lung cancer in vivo. **a** Mice were implanted subcutaneously with the indicated cell lines for ~100 mm^3^ and were treated with vehicle and erlotinib for 14 days. Each point represents the mean ± SEM of tumour volumes of three mice in each group. Tumour volume was calculated as indicated in Materials and methods. *P < 0.05 and NS, not significant. **b** The indicated tumours with vehicle and erlotinib treatment for 14 days were dissected from the surrounding tissue. **c** Immunohistochemistry assay to confirm the expression of EHD1 and CD133 in the indicated group of tumour samples. **d** The relative expression of KLF4, SOX2 and Nanog in the indicated group of tumour samples were measured by real-time RT-PCR analysis. The results are shown as the means ± SD of three independent experiments each performed in triplicate. *P < 0.05. **e** Model of the regulatory signalling networks of NF-κB/miR-590/EHD1 in erlotinib resistance and CSC properties of lung cancer. NF-κB suppresses the expression of miR-590, decreasing the targeting of the 3′ UTR of EHD1 and increasing the expression of EHD1

To further analyse the effects of NF-κB/miR-590/EHD1 on the tumourigenicity and erlotinib sensitivity of NSCLC cells, PC9/GR cells treated with PDTC were injected into BALB/c nude mice; the mice began receiving erlotinib treatment when tumour volumes reached 100 mm^3^. Compared with the control (PC9/GR/Control) group, average tumour volume of the PDTC group was significantly lower after erlotinib treatment (Fig. 7a, b). Moreover, the PC9/GR/PDTC/anti-miR-590 group showed increased tumour growth and a reduced response to erlotinib than the PC9/GR/PDTC/anti-miR-NC group (Fig. 7a, b). To investigate whether these effects can regulate CSC properties in vivo, we examined the expression of EHD1 and CD133 in the developed tumours. Consistent with our previous observations in vitro, our results showed that PC9/GR groups with PDTC treatment had a suppressed expression of CD133 and stemness-related factors, and these effects were reversed by further downregulation of miR-590 (Fig. 7c, d). These data illustrated that the NF-κB/miR-590/EHD1 axis ameliorates chemosensitivity to erlotinib by regulating CSC-like activities of lung cancer (Fig. 7e).

## Discussion

Chemotherapy resistance is a major obstacle in the clinical treatment of NSCLC. Nearly 40% of EGFR-mutant NSCLC patients cannot benefit from EGFR-TKI treatment because of acquired and intrinsic resistance to these therapeutics. The T790M or other exon 20-resistant mutations and the activation of EGFR downstream signalling contribute to the majority of EGFR-TKI resistant cases. Other recently identified mechanisms include MET amplification and EML4-ALK gene fusion^22–24^. The primary role of EHD1 appears to be regulating endocytic recycling and the return of endocytosed cargo to the plasma membrane from the ERC^25^. Moreover, EHD1 appears co-localise with EGFR, which participates in cargo internalisation and increases drug resistance. These data suggest that EHD1 might regulate EGFR-TKI resistance. Our study also confirmed that 67% of EGFR mutations in NSCLC patients with low EHD1 were more sensitive to EGFR-TKIs. More importantly, EHD1 overexpression was associated with a worse PFS in EGFR mutation-positive NSCLC patients receiving EGFR-TKIs as first-line treatment and was an independent prognostic factor for predicting outcome.

MiRNAs, small noncoding RNA molecules that suppress gene expression by binding to the 3′-UTR of target mRNAs, are involved in many biological activities, such as cancer metastasis, chemosensitivity and CSC activities^26–28^. Previous studies have reported that a high expression of miR-590-5p regulates RECK and AKT/ERK expression to induce chemoresistance and that its abnormal expression is highly associated with poor prognosis, metastasis and cancer cell proliferation^29–31^. Moreover, it was reported that miR-590-5p suppressed CSC-like properties via regulating SOX2 expression in breast cancer cells^32^. In addition, miR-590 inhibited cardiosphere-derived stem cell differentiation by targeting the TGFB pathway^33^. In this study, we whether miR-590 targeting of EHD1 and SOX2 contributes to improved erlotinib sensitivity and stem cell-like properties. In conclusion, miR-590 expression was downregulated in PC9/GR cells compared with PC9 cells, resulting in the upregulation of EHD1, which may be one mechanism by which PC9/GR cells acquire resistance to erlotinib.

Recent studies have revealed that NF-κB activation contributes to acquired EGFR-TKI resistance, and NF-κB may be a possible therapeutic biomarker for patients with EGFR-TKI resistance^34–36^. Moreover, by using specific NF-κB inhibitors, EGFR-TKI-induced cell apoptosis was increased in EGFR mutant cells^37^. Consistent with these studies, we showed that NF-κB suppresses the miR-590 level and promotes the upregulated expression of EHD1 in erlotinib-resistant cells, and the inhibition of NF-κB enhanced erlotinib sensitivity in vitro and in vivo, suggesting that novel dual inhibition co-targeting of EGFR and NF-κB might be applied in EGFR-mutant NSCLC patients to improve survival. CSCs are responsible for self-renew, tumourigenesis and chemotherapy resistance^38^. In addition, several studies have demonstrated that NF-κB increases stem cell-like properties and drug resistance via MDR1, which has been reported to increase CSC stemness and markers contributing to TKI resistance and metastasis in lung cancer^39–41^. The expression CD133 and stemness-related genes, such as Klf4, Sox2, and Nanog have been shown to have an important role in cell self-renewal and in CSC features in NSCLC^42,43^. We have provided evidence that CD133 expression is positively associated with EGFR-TKI resistance and high levels of EHD1 in patients, and NF-κB/miR-590/EHD1 acts as a novel mechanism, promoting EGFR-TKI resistance via enhanced cancer CSC-like properties in vitro and in vivo.

Collectively, our data suggest that NF-κB/miR-590 increased EHD1 expression to increase stem cell-like properties and EGFR-TKI resistance in lung cancer, suggesting that EHD1 may be an independent prognostic marker in lung cancer and that the NF-κB/miR-590/EHD1 signalling pathway might be a potentially effective therapy for overcoming EGFR-TKI resistance.

## Electronic supplementary material

Figure S1(TIF 821 kb)

Supplementary Figure Legend(DOCX 18 kb)
