# Supplementary figures and images for: NF-κB-driven improvement of EHD1 contributes to erlotinib resistance in EGFR-mutant lung cancers
Source: Cell Death Dis. 2018 Mar 16;9(4):418. doi: 10.1038/s41419-018-0447-7 (PMC5856828; doi:10.1038/s41419-018-0447-7)

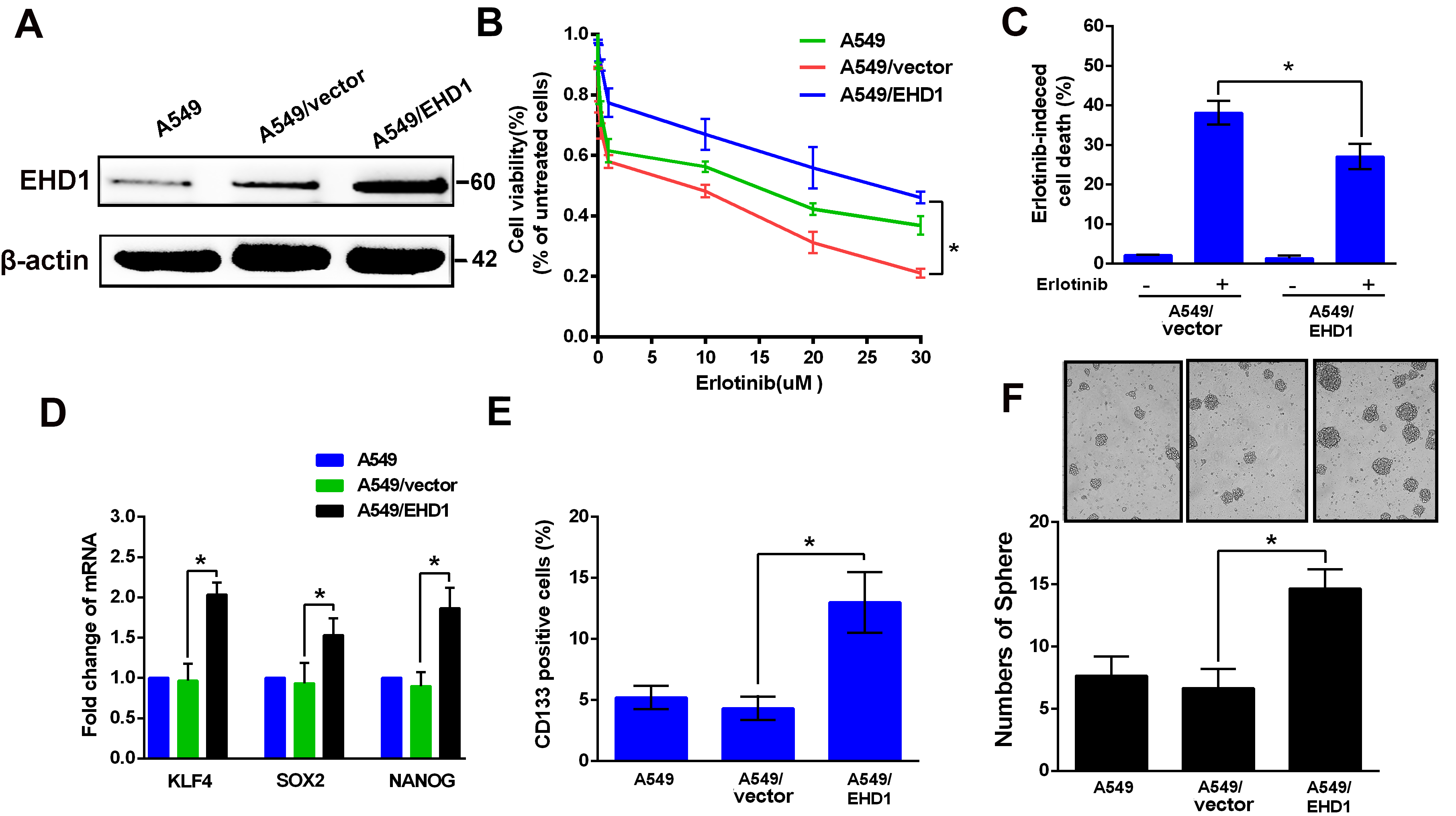

Supplement: Supplementary file 1 — Figure S1(TIF 821 kb) [file 41419_2018_447_MOESM1_ESM.tif]
