# Supplementary material for: NF-κB-driven improvement of EHD1 contributes to erlotinib resistance in EGFR-mutant lung cancers
Source: Cell Death Dis. 2018 Mar 16;9(4):418. doi: 10.1038/s41419-018-0447-7 (PMC5856828; doi:10.1038/s41419-018-0447-7)
Supplement: Supplementary file 2 — Supplementary Figure Legend(DOCX 18 kb) [file 41419_2018_447_MOESM2_ESM.docx]

**Supplementary Figure Legend**

**Figure. S1.** Increased EHD1 suppresses erlotinib-induced cell death. (A) Western blot analysis of EHD1expression in A549, A549/vector, and A549/EHD1 cells. (B) The indicated concentrations of erlotinib were added to these cells; cell viability was measured by CCK assay, and (C) cell apoptosis was detected by flow cytometric analysis. (D) The effects of EHD1 expression mediated mRNA expression of KLF4, SOX2 and Nanog. Total RNA was harvested for the analysis of mRNA by real-time RT-PCR. (E) The effects of EHD1 expression mediated CD133+ cells. Cells were harvested for flow cytometric analysis to detect CD133+ cells. (F) The graph indicated the differences in the sphere numbers per microscopic field at 100×magnification (Upper) and that was calculated (Lower). The results are shown as the means ± SD of three independent experiments, each performed in triplicate. *P < 0.05.
